# Péritonite aigue généralisée par perforation utérine post abortum à propos d'une observation

**DOI:** 10.11604/pamj.2016.24.98.9307

**Published:** 2016-05-27

**Authors:** Ibrahima Ka, Papa Saloum Diop, Amadou Bocar Niang, Alioucoly Faye, Jean Marck Ndoye, Babacar Fall

**Affiliations:** 1 Service de Chirurgie Générale, Hôpital Général de Grand-Yoff, Dakar, Sénégal

**Keywords:** Avortement clandestin, perforation utérine, péritonite, Clandestine abortion, uterine perforation, peritonitis

## Abstract

L'avortement provoqué clandestinement est responsable d'une importante mortalité maternelle. Nous rapportons un cas de péritonite par perforation utérine post-abortive. Il s'agissait d'une patiente de 25 ans reçue pour des douleurs abdomino- pelviennes, vomissements et diarrhée, dans un contexte d'aménorrhée de 12 semaines. L'examen à l'admission retrouvait un tableau de péritonite asthénique. L'exploration chirurgicale notait une péritonite aigue généralisée secondaire à une perforation du dôme utérin, avec collection purulente de 1500 cc; anses grêles dilatées et multiples fausses membranes. Le geste chirurgical: aspiration du pus, toilette péritonéale; suture utérine, drainage.les suites opératoires étaient simples, le retour à domicile de la patiente a été autorisé à J 15.

## Abstract (en)

Clandestine abortion is known to be a major contributor to maternal mortality. We report a case of a 25-year old patient in her 12th week of amenorrhea with peritonitis due to uterine perforation following abortion, admitted with abdomen and pelvis pain, vomiting and diarrhea. Clinical examination on admission showed asthenic peritonitis. Surgical exploration showed widespread acute peritonitis secondary to a perforation of the uterine dome, with collection of 1500 cc of purulent material, dilated bowel loops and multiple false membranes. Surgery: pus aspiration, peritoneal lavage; uterine suture, drainage. The postoperative course was uneventful, the patient was discharged after 15 days.

## Introduction

L'avortement provoqué clandestin reste un véritable problème de santé publique dans les pays en développement. Cette pratique non autorisée dans ces pays est responsable d'une importante mortalité maternelle [1]. La péritonite par perforation utérine reste une complication fréquente des avortements clandestins. Nous rapportons un cas de perforation utérine avec péritonite aigue généralisée.

## Patient et observation

**Figure 1:**
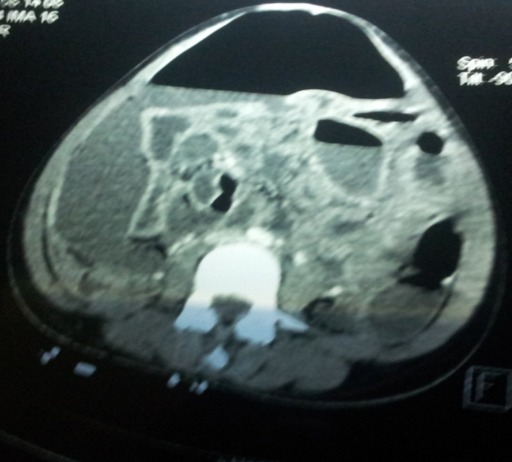
TDM abdomino-pelvienne: (1) collection purulente; (2) pneumopéritoine massif

**Figure 2:**
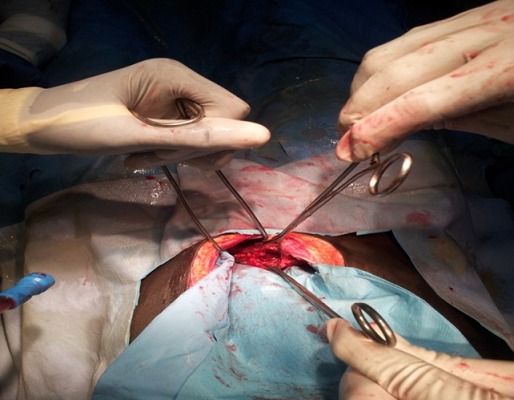
Vue opératoire, perforation du dôme utérine (flèche)

Il s'agit d'une patiente de 23 ans; 3 gestes; 3 pares, sans antécédents pathologiques particuliers reçue aux urgences chirurgicales de l'Hôpital General de Grand-Yoff pour douleurs abdomino-pelviennes, vomissements et diarrhée aigue sur aménorrhée de 12 semaines. L'examen clinique notait une patiente en mauvais état général; muqueuses conjonctivales peu colorées. L'examen physique de l'abdomen notait une distension abdominale diffuse avec défense généralisée, les touchers pelviens étaient douloureux. La biologie retrouvait un taux d'hémoglobine a 10.4 g/dl; une hyperleucocytose à 26700 à prédominance neutrophile; une CRP à 96 mg/L. le scanner abdomino- pelvien montrait un épanchement péritonéal de grande abondance et un pneumopéritoine massif (Figure 1). L'exploration chirurgicale notait une péritonite aigue généralisée secondaire à une perforation du dôme utérin (Figure 2), avec collection purulente de 1500 cc; des anses grêles dilatées et multiples fausses membranes. Le geste chirurgical consistait en une aspiration du pus, d'une toilette péritonéale; d'une suture utérine, et d'un drainage. Les suites opératoires étaient simples, le retour à domicile de la patiente a été autorisé à J 15.

## Discussion

L'avortement provoqué clandestin n'est pas une pratique rare dans les pays en voie de développement. Au Gabon, il représente 87,1% des avortements [2]. A Madagascar, sur les péritonites survenant dans le post-abortum, la moyenne d’âge des patientes était de 25,05 ans dont 32% étaient nullipares, 38% concubines et 24% des mères célibataires [3]. Ralisata a retrouvé un âge moyen similaire et des patientes paucipares [4]. L’âge moyen des grossesses au moment de l'acte de 11 semaines d'aménorrhée dans la littérature [3], était de 12 semaines chez notre patiente. Notre patiente était issue d'un milieu social défavorisé, mère 3 enfants à l’âge de 25 ans avec un époux qui vit dans un autre pays; une grossesse survenant en l'absence de l’époux est un facteur incitant à la pratique de l'avortement. Le délai entre l'acte abortif et la péritonite chez notre patiente confirme les données de la littérature [3] qui est de 4 à 40 jours avec une moyenne de 12 jours. Dans une série sénégalaise, Cissé retrouve un délai moyen de consultation était de 7 jours [5]. Le scanner nous permit de poser le diagnostic de péritonite aigue généralisée sans par ailleurs pouvoir déterminer la cause exacte. Le pneumopéritoine n’étant point lié à une perforation digestive mais plutôt à une surinfection anaérobie. Dans d'autres pays africains l’échographie reste l'imagerie la plus utilisée [3]. En Afrique, les méthodes abortives utilisées sont souvent grossières et traumatisantes [5, 6]. Le moyen le plus utilisé est la sonde intra-utérine [3, 4, 7]. Pour Cissé, les manœuvres endo-utérines avaient été effectuées avec des objets traumatisants (sondes métalliques, bout de bois) [5]. Les lésions intestinales au cours des avortements provoqués clandestins sont relativement fréquentes et de plus en plus rapportées [8, 9]. Dans la majorité des cas, le segment intestinal lésé est découvert au cours d'une laparotomie pour péritonite post-abortum [10, 11]. La complication nécessite pour se produire une perforation utérine ou plus rarement une perforation du vagin principalement le cul-de-sac de Douglas [10]. Chez notre patiente la perforation siégeait au dôme de l'utérus; sans perforation intestinale associée. Toutefois, 4 cas de péritonite par perforation utérine ont été enregistrés parmi une série continue de 101 avortements provoqués clandestins compliqués traités au niveau de la Clinique Gynécologique et Obstétricale (CGO) du Centre Hospitalier Universitaire de Dakar [5]. Dans 3 cas, les suites opératoires étaient compliquées, une fois d'une suppuration pariétale, une fois d'une péritonite secondaire ayant nécessité une ré-intervention et une fois d'un infarctus iléomésentérique responsable d'un décès maternel [5]. La péritonite secondaire à un avortement provoqué clandestin est une pathologie grevée d'une lourde mortalité. A Madagascar, 15,1% ont succombé dans les sept jours suivant leur admission à l'hôpital, et 6,7% avant l'intervention chirurgicale, soit un total de 21,8% de mortalité. Tous ces décès ont été dus à un choc septique [3].

## Conclusion

Les complications des avortements provoqués clandestins sont de plus en plus fréquentes. Nous avons rapporté un cas d'une perforation utérine compliquée d'une péritonite aigue généralisée. Cette observation met en exergue les difficultés de prise en charge des complications liées à une pratique qui reste encore clandestine dans nos pays, mais qui n'en demeure pas moins fréquente.
